# Goal-oriented requirements engineering: an extended systematic mapping study

**DOI:** 10.1007/s00766-017-0280-z

**Published:** 2017-09-14

**Authors:** Jennifer Horkoff, Fatma Başak Aydemir, Evellin Cardoso, Tong Li, Alejandro Maté, Elda Paja, Mattia Salnitri, Luca Piras, John Mylopoulos, Paolo Giorgini

**Affiliations:** 1 Chalmers and the University of Gothenburg, Gothenburg, Sweden; 2 Utrecht University, Utrecht, Netherlands; 3 University of Trento, Trento, Italy; 4 Beijing University of Technology, Beijing, China; 5 University of Alicante, Alicante, Spain; 6 University of Ottawa, Ottawa, Canada

**Keywords:** Goal model, Systematic mapping study, Goal-oriented requirements engineering, GORE

## Abstract

Over the last two decades, much attention has been paid to the area of goal-oriented requirements engineering (GORE), where goals are used as a useful conceptualization to elicit, model, and analyze requirements, capturing alternatives and conflicts. Goal modeling has been adapted and applied to many sub-topics within requirements engineering (RE) and beyond, such as agent orientation, aspect orientation, business intelligence, model-driven development, and security. Despite extensive efforts in this field, the RE community lacks a recent, general systematic literature review of the area. In this work, we present a systematic mapping study, covering the 246 top-cited GORE-related conference and journal papers, according to Scopus. Our literature map addresses several research questions: we classify the types of papers (e.g., proposals, formalizations, meta-studies), look at the presence of evaluation, the topics covered (e.g., security, agents, scenarios), frameworks used, venues, citations, author networks, and overall publication numbers. For most questions, we evaluate trends over time. Our findings show a proliferation of papers with new ideas and few citations, with a small number of authors and papers dominating citations; however, there is a slight rise in papers which build upon past work (implementations, integrations, and extensions). We see a rise in papers concerning adaptation/variability/evolution and a slight rise in case studies. Overall, interest in GORE has increased. We use our analysis results to make recommendations concerning future GORE research and make our data publicly available.

## Introduction

The quality of a software system critically depends on the degree to which it fulfills its requirements. Such requirements can be elicited, modeled, and analyzed as stakeholder goals. The field of goal-oriented requirements engineering (GORE) has emerged in order to create and study various methods which approach RE from a goal-oriented perspective. Typically, within GORE, goals are elicited and conceptualized in terms of some form of model. Goal models have been used as an effective means for capturing the interactions and trade-offs between requirements, but they have been applied more broadly to advance the state of software adaption, security, legal compliance, and business intelligence, among other areas.

In this work, we aim to understand the landscape and status of existing work in GORE at a high level of abstraction. In a recent RE meta-survey, Bano et al. have pointed out that there has yet to be a systematic literature review of GORE publications [3]. Although a few GORE reviews exist, e.g., [1, 18], they are focused on sub-topics or frameworks within GORE, and not the area in its entirety. Based in part on these past surveys and on the experiences of the authors, it is apparent that there is a large body of GORE-related publications available. Thus, it becomes important to provide a broad overview of this work, helping to reflect on the state-of-the-art and guide future research.

In this work, we produce a systematic mapping study (SMS) summarizing publications falling under the scope of our study without considering their quality [26, 34]. This SMS can be beneficial for several types of readers. For researchers interested in GORE, the map helps to build upon existing work, avoiding the proverbial “reinvention of the wheel,” helping to understand trends, and guiding efforts in new directions. For practitioners, this map offers ideas on the most prominent GORE methods and frameworks, including pointers to work containing further details.

As per available SMS guidelines [26, 34], we focus our investigation on a set of particular research questions. Broadly speaking, we are interested in mapping the space of GORE research, providing a structure and visual summary of the field as per [34]. In order to provide this structure, we classify the types of GORE publications (proposals, extensions, meta-studies, etc.), the nature of research evaluation, common topics appearing in GORE work, trends in topics, common frameworks, publication venues, citation distributions and networks, author statistics, and co-author networks. Overall, we ask whether interest in GORE is increasing or decreasing. We analyze our findings, discussing possible underlying reasons for our mapping results.

This work is an extended version of the conference paper published in [19]. We have extended our SMS in several significant ways: we provide more information on trends in paper topics, topic distribution through paper venues, citations per paper types, citations per paper topics, dominant papers within each topic, and examine the network of paper citations. We also look at the main contributors to GORE research and examine the network of co-authors. We expand our discussion to cover these new findings. More detail is provided on our process of measuring inter-coder reliability. We make a more extensive study of related work, including a more detailed consideration of the 21 meta-studies discovered as part of our SMS.

This work follows the same theme as previous work by some of our authors presented in [18, 20], but with a different focus and method. These papers provided a SMS [20] and then a systematic literature review (SLR) [18] focusing specifically on approaches which transform or map to or from goal-oriented methods, a subset of the focus of this paper. These previous surveys found papers through a mix of systematic search and reference “snowballing,” without using the number of citations as an exclusion criterion. In this work, due to our broader coverage, we use only systematic search, with a citation cutoff to manage survey size. Despite the broader scope of the current survey, because of these differing methods of finding papers, the publications included in the current survey are not a super-set of the papers in the previous surveys, i.e., most publications included in these previous surveys are not included in the current SMS. Specifically, the overlap between the 170 papers included in [20] and the 246 papers included in this survey is 29 papers, while the overlap with the 247 papers in [18] is 40.

The rest of the paper is organized as follows. We first introduce our research questions in Sect. 2 and then describe the scope, classification schema, and key terminology of our study in Sect. 3. Section 4 presents the method followed. Section 5 summarizes the results of the SMS, while Sect. 6 discusses survey results and design alternatives. Section 7 lists threats to the validity of the study. Section 8 reviews related work, while Sect. 9 offers conclusions and ideas for future work.

## Research questions

As per Petersen et al. [34], we articulate the specific research questions (RQs) guiding our SMS, including more detailed sub-questions, listed in Table 1. We include research questions covering basic bibliographic information such as venue (RQ5), citations (RQ6), authors (RQ7), and publication numbers (RQ8). Although comparatively easy to collect, such cursory information is still helpful in understanding GORE research and is common to most SMSes [34]. We also include several deeper questions. Given the wealth of GORE papers, we are particularly interested in whether a publication proposes a new method or reuses existing work, and in what way (RQ1). This helps us to gage the convergence and maturity of the field. Given the rising interest in empirical RE [9], we want to understand the type of evaluation GORE papers have applied (RQ2). We want to know for what types of problems goal models have been applied (RQ3). In several of these areas, we find it particularly useful to see trends over time (RQ1, 2, 3, 8), indicating which practices are increasing or decreasing in popularity. Finally, given the divergence in GORE methods, it is particularly interesting to know what frameworks have been used (RQ4).

It is important to note that our unit of analysis is publications, and not research approaches (e.g., frameworks such as KAOS, i*, Secure Tropos). Focus on approaches would be interesting, but is subject to much interpretation. See Sect. 6 for a discussion of alternative survey approaches.

**Table 1:** Research questions

RQ1	Types	(a) Can GORE publications be classified as particular types of papers: proposals, formalizations, meta-studies, integrations, extensions, ontological interpretations, implementations?
		(b) How has this changed over time?
RQ2	Evaluation	(a) Do GORE publications contain evaluation?
		(b) Of which type?
		(c) How has this evolved?
RQ3	Topics	(a) Which are the topics covered by GORE publications?
		(b) How have these topics evolved over time?
RQ4	Frameworks	Which goal modeling frameworks have been used in the publications?
RQ5	Venue	(a) In which venues (journals or conferences) do GORE approaches typically appear?
		(b) How are the paper topics distributed in these venues?
RQ6	Citations	(a) Which publications are most widely cited?
		(b) Are citations equally distributed?
		(c) How do they vary per citation source?
		(d) Which types of papers are the most cited?
		(e) Which topics are the most cited?
		(f) Are there dominant papers within each topic?
		(g) What does the network of citations look like?
RQ7	Authors	(a) Who are the main contributors?
		(b) What does the network of co-authors look like?
RQ8	Interest	Is interest in GORE increasing or decreasing?

## Scope

In this section, we provide definitions of key concepts used to define the scope and classification schemes of our SMS.

### Key terms

We define *goal-oriented requirements engineering* as the study or application of goal models in requirements engineering. A *goal model* is a model expressed in a goal-oriented language. Such languages include the concept of goal as a first class object, are often graphical, and come with a visual syntax (e.g., i* [50], KAOS [10]), but may also be textual (e.g., GBRAM [2]). We adopt the notion of a *language* from [16]: “a language consists of a syntactic notation (syntax), which is a possibly infinite set of legal elements, together with the meaning of those elements, which is expressed by relating the syntax to a semantic domain.” Languages can be graphical or textual, and the semantics (meaning) can be formally or informally defined.

### Inclusion and exclusion criteria

We focus our investigation on publications appearing in international journals, conferences, or symposia. Publications must be in English, in order for all our author/coders to be able to read them. We omit theses, focusing on work which has been published in international venues. Among venues, we exclude workshop publications and regional conferences, as the quality and impact of these venues can vary widely. We omit very short papers, as these papers often serve a different purpose and have a different style compared to longer publications. In order to make it feasible to process the number of papers found, we exclude papers with a low (less than 3) number of citations, and we discuss this decision in Sect. 4.4.

We include papers which deal significantly with GORE; by this, we mean that the main purpose or contribution of the paper involves GORE. If, on the other hand, the paper mentioned GORE only comparatively, or GORE was used as only a small aspect of a contribution, the paper would be excluded. These criteria were discussed at length among the paper coders, using examples to highlight borderline cases. More information about the inclusion/exclusion process is provided in Sect. 4.5. Our scoping criteria are summarized in Table 2.

**Table 2:** Publication inclusion and exclusion criteria

Inclusion criteria	Exclusion criteria
Has a significant component that deals with GORE, and	Does not significantly relate to GORE or
Is published in a conference, journal, or in/is a book, and	Is a thesis, is published in a workshop or regional conference, or
Is published in English	Is published in another language
Is more than three pages, and	Is three pages or less, or
Has been cited 3 or more times according to Scopus as of 16 Dec 2015	Has been cited less than 3 times according to Scopus as of 16 Dec 2015

Although we focus on the use of goal models in Requirements Engineering, we do not exclude those publications which are either aimed for different research fields, or which apply goal models to a new context, as long as the authors relate their work back to GORE. See the description of our search string in Sect. 4 for more information.

## Survey method

In this section, we describe the pre-SMS preparation, SMS steps, and post-SMS processing.

### Pre-SMS preparation

#### Guidelines for reading publications

It was necessary to work out clear guidelines as to how, and to what degree, to read selected publications. We needed to establish such common guidelines both for the preprocessing of papers (e.g., to calculate inter-coder reliability) and for the processing of the final paper set. Given the high level of abstraction of our SMS, it was not necessary to carefully read each paper in its entirety. Many mapping studies restrict reading to the abstract or introduction. We decided to read the title, abstract, introduction, and conclusion. The reader/tagger could optionally flip through the details of the paper, particularly section headings, to make clarifications or resolve questions. As most papers were about modeling, perusing was particularly useful to see the details of the included model(s). We were also allowed to search the paper for keywords, using a custom-made script to enable us to search for multiple keywords at once.

### Classification schemes

We endeavoured to understand GORE publications via two classification schemes. We call the process of applying these categories to papers “tagging” or “coding,” as per the typical terminology of qualitative coding or tagging, applying one or more “tags” or “codes.” The first, the *type* of paper refers to the research contributions, methods, and/or structure provided by the paper. We started with an initial conceptualization for the paper type scheme based on our knowledge of research methods and our research questions (particularly RQ1 and 2). Our scheme bears similarities to the classification scheme of Wieringa et al. [48]; however, after our experiences using this scheme in [18, 20], we designed a slightly broader, more descriptive scheme.

The second classification refers to the *topic* of the paper (e.g., scenarios, agile, NFRs), independent from the research method. In order to derive paper topic tags, we performed a grounded analysis, inspired by grounded theory [41]. We started with a set of papers we knew to be related to GORE (extracted from the related work sections of the author’s theses, covering several goal model-related topics) and then “snowballed” through the papers, following the reference links to other related papers, assigning type and topic tags to each paper, and proposing our own perceived topics. When tagging a paper, we tried to be true to the terminology used by the authors, e.g., if the authors say they extended goal models with scenarios, we would include the *extension* tag as a paper type and the *scenario* tag as a paper topic.

The tagging processes ended when we got to a set of 110 papers, adopting the set of topics generated so far. At this point, we felt the list of topics was beginning to converge. Of course, it is always possible to find further topics, and we do not claim that our final list of topics is complete. In a group discussion, we evaluated the topics, merging similar topics. This was particularly done when we found it was difficult to distinguish reliably between different topics, or the topics frequently co-occurred. We developed collective definitions for each topic and listed a set of helpful keywords. Keyword searches were intended to act as a helpful tool to supplement the manual process. We had no formal criteria for the number of keyword occurrences in order to add a tag, but left the assignment to human judgment.

The process of tagging the initial set of 110 relevant papers also helped us to refine the paper type scheme, with unclear types removed or refined. The final set of codes is found in Tables 3 and 4.

**Table 3:** Classification scheme: paper types

Types	Description
Proposal	Any publication that proposes something new, e.g., a language, extension, integration, algorithm,. New evaluations of a language or method (e.g., case studies, experiments or experience reports) would not count as a proposal. We decided not to judge the degree of novelty ourselves, as this is very subjective, only to determine whether something new was proposed or not
Formalization	If the publication contains axioms, some formal logical language, relating to the proposal, it has a formalization. We particularly looked for logical operators (e.g., $\lnot$, $\vee$, $\Rightarrow$). Again, it was not our task to judge the quality of the formalization, only if some formalization was present. We did not count pseudocode as a formalization
Meta-study	Publications which provided a significant overview of existing work or a study of existing research. Examples include surveys, reviews, and sometimes vision papers. We looked for publications that emphasized an analysis of existing work beyond the typical related work section
Implementation	Publications that mention the development of a tool or implementation which facilitates the contribution of the work. We gave no credit for being in the process of building a tool, or providing pseudocode without an implementation. The tool did not have to be implemented by the paper authors
Integration/transformation/mapping	The category was assigned if the publication contribution described two different, distinct, named things, one of which was a goal model, and this goal model was integrated, transformed, or mapped to the other thing
Extension	Publications which focus on some concept(s) which is not a named language or method being added to goal model (e.g., capabilities, commitments)
Ontological interpretation	A publication which maps ontologies onto some aspects of goal models. Formalizations are considered interpretations but not ontological interpretations
Evaluation: bench mark	Evaluating a contribution using an established and shared measure or example
Evaluation: case study	The publication includes a case study which evaluates the contribution. Whether the case study is a case study or only an illustrative example depends on depth and realness. If the case is detailed, real, or if there is more detailed information available in another source, typically it is a case study. The authors do not have to have conducted the case study themselves, but could also use data from an existing case study
Evaluation: controlled experiment	The publication includes a controlled study in order to evaluate their contribution
Evaluation: questionnaire	The evaluation includes a questionnaire collecting answers from some target group and evaluating the results
Evaluation: scalability	The publication evaluates the performance of all or part of the contribution; this could include computational scalability, model size, or scalability in terms of human effort

**Table 4:** Classification scheme: paper topics

Types	Description	Keywords
Adaptation, variability, and evolution	The paper deals significantly with adaptation, variability, evolution or automatically changing systems and/or models	Adapt* (variations of adaptation), variability, evolution, autonomic
Agents	The publication uses or talks about agents or actors fairly significantly	Agent, actor
Aspects	Work that uses or talks about software aspects fairly significantly	Aspect
Business intelligence/modeling	The publication focuses on the use of analytics, software, or data to drive business decisions. Data are often connected to enterprise or business modeling, showing how the business works	Business intelligence, business modeling, KPI, indicator, enterprise modeling, strategic management
Compliance	The publication deals with evaluating compliance with laws, regulations or policies	Compliance, law, policy, regulation
Conflicts	Publications involving all aspects of conflicts, including identification, management, discovery, and resolving	Conflict
Requirements engineering	The paper focuses on, or is in the field of RE	Requirements engineering, RE, requirements
Early requirements engineering	Publications dealing with the very early stages of RE, often with social, vaguely defined goals	Early, early RE, early requirements, early requirements engineering
Model-driven development	Publications which focus significantly on some form of model-driven-*. The authors should use these words specifically	MD* (MDD, MDE, MDA), model-driven
Non-functional requirements	The paper is primarily about NFRs or softgoals, using them significantly in the model, process, or analysis	Softgoals, NFR, non-functional
Systematic reasoning	The work contains algorithmic or mathematical analysis of a model to answer some question(s) or find one or more properties. This can be formal, qualitative, quantitative, automated, interactive, or manual, as long as it is systematic and repeatable. The reasoning should be demonstrated in the paper, not just have the potential to do some reasoning	Reasoning, analysis, automated, propagation, evaluation, metrics
Privacy/security/risk/trust	The publications deals significantly with privacy, security, risk, and/or trust	Privacy, security, risk, trust
Architecture	The paper discusses or focuses on some type of architecture, either of software, systems or of a business	Architecture
Patterns	The publication discusses or uses in some significant way some type of pattern: software, design, requirements, etc.	Pattern
Agile	A paper discusses, uses or applies all or part of an agile method	Agile, scrum, lean, extreme, XP
Scenario	The paper uses scenarios/use cases/sequences as a requirement engineering technique in conjunction with goal modeling	Scenario, sequence, use case

### Inter-coder agreement

After deriving an initial set of tags, it was necessary to evaluate how consistently the coders could apply the type or topic tags. We performed two rounds of inter-coder reliability (ICR) tests. For these rounds, we used papers randomly selected from the goal model-related bibliographies of the theses of the first seven authors. As we estimated our final set of papers would be approximately 300, we chose a set of 30 papers potentially related to GORE, making up about 10% of the final size, a recommended minimum as per ICR-related literature [29].

The initial team of paper taggers was made up of seven postdoctoral fellows and graduate students with some association to the University of Trento and some experience with goal modeling. In the first round of ICR testing, all seven coders coded a set of 30 potential GORE papers; however, two coders did not finish in time for us to take measurements. We evaluated ICR on the types and topic tags using Krippendorff’s alpha [28], which indicates our coding consistency per code across all 30 papers. Each paper type or topic mapped to a single code treated as a binary variable, with a yes/no decision from each coder. Krippendorff’s alpha is recommended for multiple coders using multiple codes and gives us the benefits of showing specifically which codes we perform well or poorly on; in other words, we are able to evaluate agreement on the codes, not the coders, which is more useful given the presence of many codes in our SMS. Here we aim for an agreement level minimum of 0.67, ideally greater than 0.80, as per [28].

The mean, median, min and max ICR scores for the first round, round 1a, using five coders is shown in the first row of Table 5. Detailed scores for each tag can be found in the second column of Tables 6 and 7. Note that ICR scores measure agreement accounting for chance, so a score of zero does not mean we did not agree, but that we are as accurate as choosing values randomly. As our agreement on several tags was low, after discussing the meanings of the tags, we decided to better emulate the final process by revisiting our tags on the same papers in set pairs of two (round 1b), with a total of three groups of two coders (omitting one coder). Interestingly, this lowered our scores. Although the groups converged within themselves, there was still much divergence between groups.

**Table 5:** Krippendorff’s alpha ICR results

Round	Pub#	Paper types				Paper topics			
		Mean	Med	Min	Max	Mean	Med	Min	Max
1a	30	0.71	0.76	0.48	0.92	0.52	0.54	0.19	0.88
1b	30	0.62	0.66	0.08	1.0	0.42	0.40	0	1.0
2	25	0.74	0.79	0.5	0.88	0.63	0.61	0.19	1.0

**Table 6:** Krippendorff’s alpha detailed ICR results for paper types

Paper types	Round 1a	Round 1b	Round 2
Proposal	0.92	1	0.50
Formalization	0.81	0.80	0.74
Meta-study	0.88	0.84	No data
Implementation	0.72	0.66	0.81
Integration	0.76	0.81	0.84
Extension	0.80	0.61	0.57
Illustrative example	0.48	8.25	Dropped
Ontological interpretation	Not exist	Not exist	No data
Evaluation bench mark	Not exist	Not exist	0.79
Evaluation case study	0.80	0.74	0.75
Evaluation controlled experiment	0.59	0.49	No data
Evaluation questionnaire	Not exist	Not exist	No data
Evaluation scalability	0.53	0.39	0.88

**Table 7:** Krippendorff’s alpha detailed ICR results for paper topics

Paper topics	Round 1a	Round 1b	Round 2
Adaptation, variability, and evolution	0.80	0.49	0.83
Agents	0.53	0.31	0.44
Aspects	No data	No data	1
Business intelligence	0.79	0.79	0.59
Business modeling	0.62	0.64	Merged
Compliance	0.70	1	0.19
Conflicts	0.29	0.14	0.59
Early requirements engineering	0.61	0.45	0.41
Model-driven development	0.20	0	0.71
Non-functional requirements	0.32	0.45	0.67
Privacy	0.19	0	Merged
Reasoning	0.54	0.24	0.63
Requirements engineering	0.54	0.36	0.59
Security, privacy, and risk	0.88	0.84	0.87
Social modeling	0.29	0.18	Dropped

We repeated the process in round two for a different set of 25 papers randomly selected from the same sources, selecting slightly less papers this time due to time constraints. Before performing this second round, we took several actions: (1) we had extensive discussions on the meanings of tags with scores <0.8, coming up with shared text definitions for all tags, (2) we dropped and merged some tags which caused confusion, (3) we dropped a coder with background less related to GORE, leaving us with six coders in total, and (4) we tagged individually but had all codes checked by a second, rotating person. For the last point, after each coder had coded each paper, we assigned a second coder to each paper, such that each coder would be a pair with each other coder the same amount of times. The second coder checked the tags of the first, and disagreements were discussed. The summary results for round 2 are shown in the third row of Table 5, while more details are found in Tables 6 and 7.

Although scores improved overall, some of the tags still had less than optimal agreement. We went through a second round of group discussions, refining definitions, changing, and adding some further tags. Due to time constraints, we opted not to do yet another round of ICR coding and testing. As this process had already taken six months (see Sect. 6 for a discussion of why), we were not convinced that extra time would be worth the possible increase in scores. ICR scores are discussed further in Sect. 7.

Tables 6 and 7 also show the evolution of our tags. A few tags were added, merged, or removed between rounds (not exist, merged, dropped). Typically, this was done after much discussion among the coders to deal with tags which were ambiguous, similar, or which were particularly difficult to tag consistently, e.g., illustrative example. In some cases, there were no data for a particular tag, as that topic or paper type did not appear in our subset of papers (no data).

As shown in Tables 3 and 4, a few final tags were added before we embarked on the final coding process (e.g., architecture, patterns, agile). As these tags do not have accompanying ICR evaluation, their reliability may be questionable. However, we felt they occurred frequently enough to include and had discussions ensuring we agreed on their meaning.

### Systematic search

After snowballing in order to derive topic tags (Sect. 4.2), it was our opinion that the process of finding a set of papers was not converging—after processing 110 papers, we were still finding an increasing number of papers with few overlaps. In order to make the paper search more manageable, and to reduce potential bias in selecting among candidate publications, we moved our focus from snowballing to systematic search. We discuss this choice further in Sect. 6. We evaluated various potential sources, including Google Scholar and Web of Science, and we decided to perform our search through Scopus, as it covers major publishers in RE (ACM, Springer, IEEE) and is more inclusive than Web of Science, but less inclusive than Google Scholar, which may include many non-peer-reviewed papers such as technical reports. Note that although we perform our publication search using only Scopus, we extracted and compared citation data from Scopus, Google Scholar, and Web of Science.

We derived our search string from our research questions, searching the title, abstract, and keywords for : (“goal-oriented” OR “goal model” OR “goal modeling” OR “goal modelling”) AND “requirements,” limiting the search to conference proceedings, book chapters, (journal) articles, or articles in press. As of 16 Dec 2015, we found 966 results.

It was clear that it was not feasible to evaluate all 966 papers; furthermore, we found that many papers had a very small number of citations according to Scopus (394/966 papers, 41%, had 0 citations). We chose to evaluate all publications having three or more citations according to Scopus, evaluating a total of 350 publications. During our publication processing (described in the next section), we found 104 papers that were out of the scope according to our criteria, ending up with 246 papers included in our study.

### Publication processing

In order to apply the inclusion and exclusion criteria, collect relevant bibliometric information, and add appropriate tags to our start set of 350 papers, we adopted the following process: we divided the papers up into six roughly equal groups, sorting by number of citations then assigning every sixth paper to a group. Each group was given to a single coder (one of our authors) to process. As described in Sect. 4.3, the ICR process resulted in a set of six coders. This means that each coder had to process about 60 papers.

When processing a paper, we first used the inclusion and exclusion criteria to determine whether the paper was in or out. If the paper was in, we collected basic bibliometric information for each included publication. Some of this information was extracted automatically from Scopus, while the rest was added (or corrected) by hand. For each included publication, we kept track of: the paper title, authors with their affiliations and countries, venue, type of venue, year, number of citations (according to Scopus, Google Scholar, and Web of Science), number of pages, and GORE framework (e.g., i*, KAOS). In cases when the GORE framework was not clear or multiple frameworks were applied, we used the tags “general” or “multiple,” respectively. We also added tags for paper types and topics as per the scheme in Sect. 4.2. In several places in our data-collection form, coders could indicate the presence of “doubts” or issues, for example, if they were unsure about exclusion of a paper or a particular code.

When this process was complete, each paper was reassigned to a second coder, for a cross-check. We assigned the papers such that every coder was checking a roughly equal number of papers coded by each other coder. The second coder reviewed papers inclusion/exclusion and tags, raising issues in various fields when they thought a code was missing or incorrect. Issues raised by both the first and second coders were stored in the database. These issues were discussed and resolved, first among the pair of coders, and in case of continued disagreement by the entire group of 6 coders. Overall, we found and resolved 182 issues concerning 124 out of 246 papers.

Finally, we performed a round of data cleaning to check and resolve missing fields or any remaining issues. The first review stage took our coders about a month, while the second round took about three weeks. It took each coder anywhere from 10 to 30 min to process each publication.

## Mapping study results

We present the data for each of our RQs with an emphasis on visual maps and graphs, as is recommended for SMSes [34]. We make our data and category descriptions publicly available.^1^

### RQ1: Paper types

**Fig. 1:**
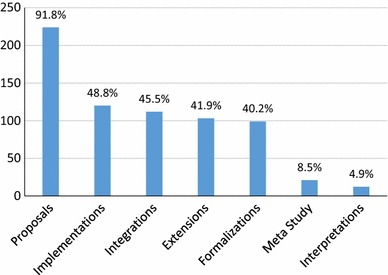
Count of paper types (**RQ1(a)**)

We summarize the number of classifications for our 246 papers in Fig. 1, answering **RQ1(a)**. Our classifications are overlapping, we have 938 type tags over 246 papers, an average of 3.8 type tags per paper. We can see that nearly all (91%) of papers propose something new, while about 46% of papers include some form of integration/transformation/mapping, and about 42% include some form of extension. Around 40% of the publications offer some sort of formalization, and nearly half, 49% offer some sort of implementation. Ontological interpretations are relatively rare (5%), as are meta-studies (9%). Overall, the focus seems to be on proposing independent new approaches, while only making extensive use of past approaches in less than half of the included papers.

**Fig. 2:**
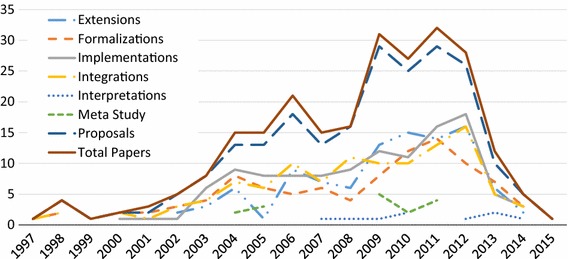
Paper types per year (**RQ1(b)**)

We can gain further insights by tracking these data over time (Fig. 2), answering **RQ1(b)**. The top line shows the number of papers per year, as a comparison. We remind the reader that publications can be tagged with more than one type, and thus, the sum of the line heights will be higher than line for papers per year. This holds for all our graphs looking at trends over time. For this and any other graph showing information per year, we must account for the fact that our mapping includes only those publications with more than three citations. Thus, we have a bias toward older publications, while newer publications are less likely to be included. This must be accounted for when considering the drop in all data from 2013 to 2015.

We see the proposals hold steady with the total number of papers, while most other types of papers hold at about half the total number of papers. Implementations seem to be on the rise, with the number of integrations and extensions also appearing to rise slightly, all relative to the number of papers. This breakdown gives a slightly more optimistic view, with incorporation of past approaches seemingly on the rise.

### RQ2: Evaluation

156 (63.4%) of GORE papers in our SMS contain some form of evaluation (**RQ2(a)**). Overall, 53% of the 246 papers contain a case study, as per our tag definition, 27% some evaluation of scalability, 7% a controlled experiment, 7% questionnaires, and 4% contain some type of benchmark (**RQ2(b)**). Recall that papers can have multiple tags, and thus, the percentages do not sum to 63.4%.

**Fig. 3:**
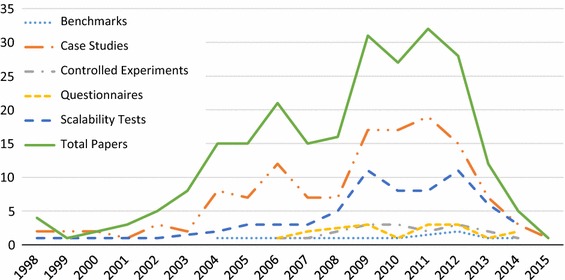
Evaluation types per year (**RQ2(c)**)

The evolution of these tags over time is shown in Fig. 3, answering (**RQ2(c)**). In general, the rise and fall of each type of evaluation follows the pattern of number of papers per year. We can see some low points in the evaluation of scalability relative to the number of papers, while empirical studies other than case and scalability studies are low overall. It appears the use of case studies may be on a slight rise, as the slope of the number of papers is steeper than the case study slope beyond 2012. For example, in 2008 44% of papers have case studies, compared to 54% in 2012. Future analysis is needed to determine whether this trend continues to hold.

### RQ3: Paper topics

**Fig. 4:**
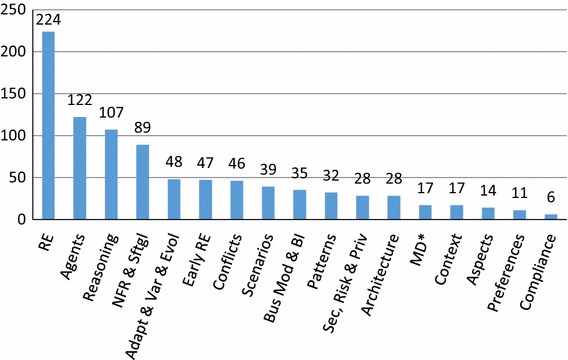
Total paper topics (**RQ3(a)**)

Figure 4 shows the breakdown of paper topics (**RQ3(a)**), and we have 910 topics over 246 papers, with an average of 3.7 topics per paper. We can see that most papers (91%) involve RE, unsurprisingly given our search string, but 9% of papers were significantly out of the RE field. Other popular topics include agents (50%), reasoning (43%), and NFR/softgoals (36%).

**Fig. 5:**
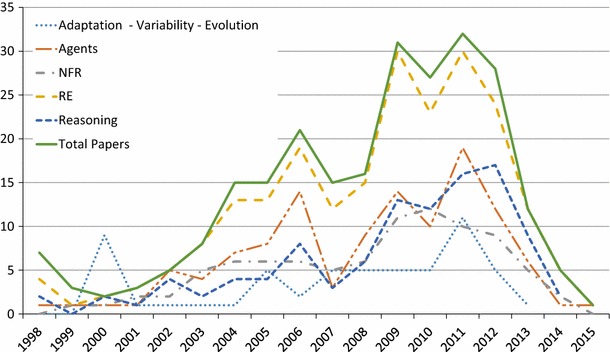
Top five total paper topics per year (**RQ3(b)**)

**Fig. 6:**
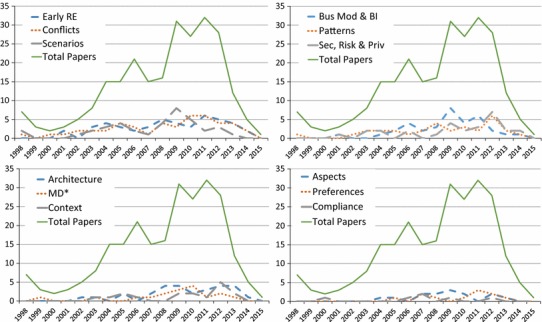
Remaining paper types per year (**RQ3(b)**)

In response to **RQ3(b)**, we show a breakdown of the top five topics per year in Fig. 5, starting from 1998. The remaining 12 paper topics per year are shown in Fig. 6. Examining trends in the popular topics in Fig. 5, the focus seems to rise and fall with the general number of papers, with a few exceptions. Interest in reasoning seems to have decreased relatively between 2009 and 2011, but seems to have increased relatively in 2012. Interest in adaptation/variability/evolution has increased recently relative to other topics, possibly accounting for the latest spike in overall GORE papers. NFR/softgoals interest appears to be decreasing.

Looking at the remainder of the topics in Fig. 6, we can see spikes in interest in scenarios around 2009–2010, in business modeling and BI from 2009 to 2012, in security, privacy, and risk around 2012–2013, and in context around 2012. Interest in topics such as scenarios, business modeling and BI, and MD* seems to be dropping recently compared to the total number of papers, while topics like early RE, conflicts, patterns, security, privacy and risk, and architecture appear to hold steady. Given the low paper counts, no other patterns can be obviously picked out from the data.

### RQ4: GORE frameworks

**Fig. 7:**
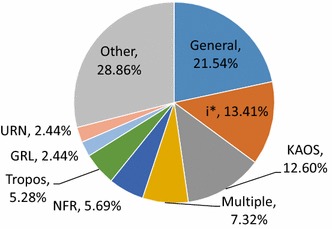
Frameworks used in 246 publications (**RQ4(a)**)

Figure 7 shows the GORE frameworks used in our included publications. As only one framework tag was recorded for each paper, we can view these results in a pie chart. We can see that although KAOS and i* appear in nearly the same number of publications (13%), the most popular choice is to use goal modeling in general, without committing to a particular framework. It is also fairly common (7%) to significantly use multiple frameworks in one paper. We note that nearly 29% of papers fall under “Other,” introducing their own framework with a new name and few subsequent publications. After KAOS, the next most popular named framework appearing in Fig. 7 is the NFR Framework, Tropos, GRL, and URN. After this, the next most frequent frameworks (shown as part of “Other” in Fig. 7) are Archimate with 1.6% of publications, GSRM, Techne, and AoURN with 1.2%, REF, Secure Tropos and ARE with 0.8%, then 52 other named frameworks with one publication (0.4%) each.

### RQ5: Venue

**Fig. 8:**
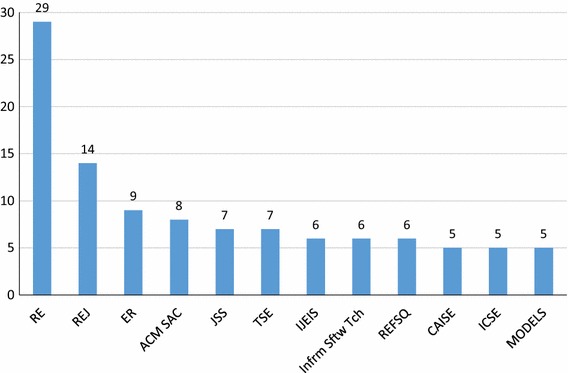
Top 12 publication venues (**RQ5(a)**)

Our SMS found a total of 111 unique venues. We show the top 12 publication venues in Fig. 8, each with five or more publications (**RQ5(a)**). We can see that the *RE conference* dominates, followed by *REJ*, then other conferences and journals with roughly equal paper numbers. One hundred and seven out of 246 (43%) publications in our SMS appear in one of these top 12 venues, meaning that the spread of publication venues is still quite wide. This can make it difficult to consolidate and share GORE knowledge, but also helps to demonstrate the uses of GORE beyond the RE community.

We can also examine the distribution of GORE paper topics in various venues. These results are potentially useful not only to understand where publishing efforts in various topics have focused, but also to help future authors consider potential publication venues given their particular topics.

**Fig. 9:**
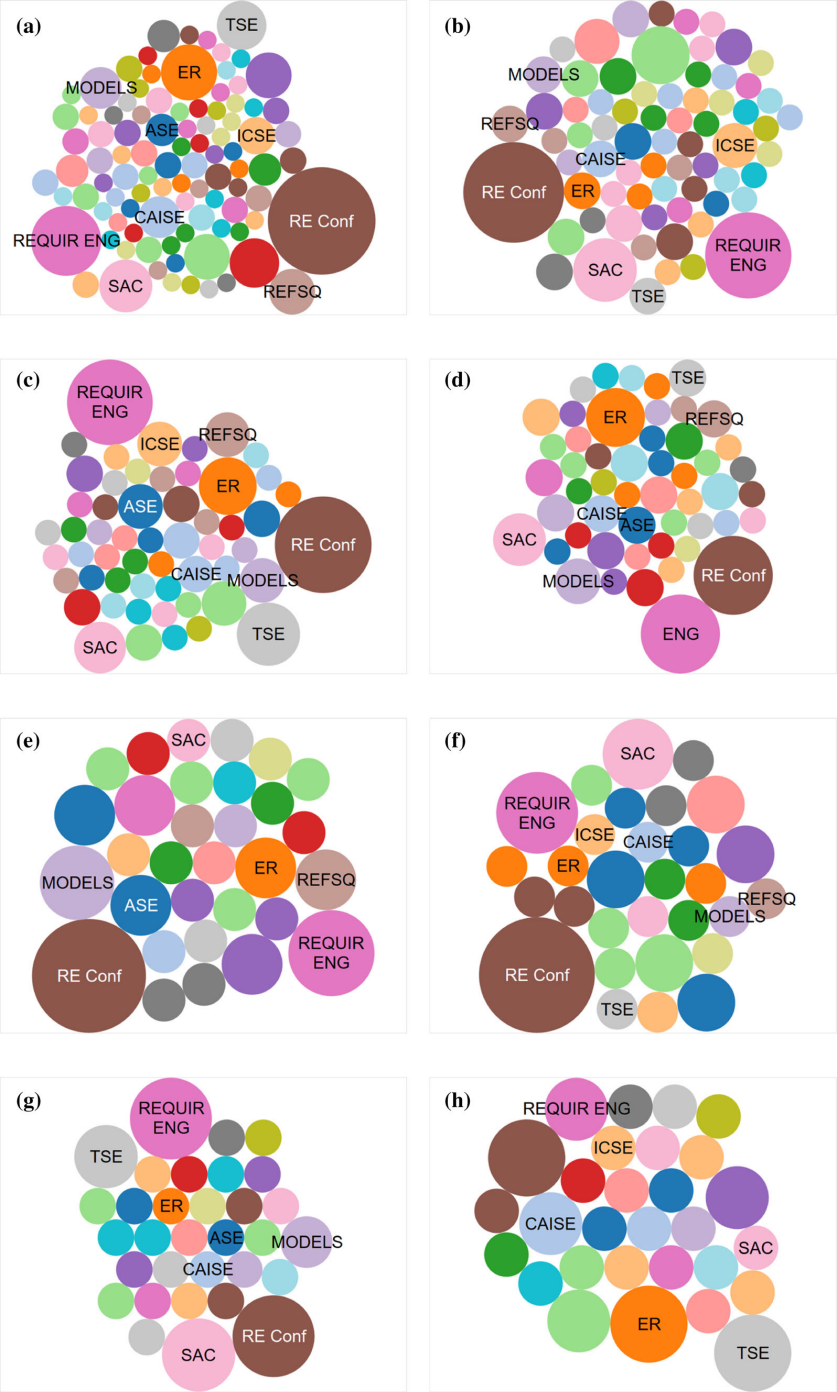
Venue occurrence for the most popular paper topics **RQ5(b)**. **a** RE, **b** agents, **c** reasoning, **d** NFR and softgoals, **e** adapt, var and evol, **f** early RE, **g** conflicts, and **h** scenario

We focus on the eight topics which occur most frequently in our SMS. For each topic, we count the number of papers tagged with that topic in the various venues, using the counts to make bubble charts, shown in Fig. 9. Here, the size of the bubble is relative to the number of papers with the particular tag in the venue. The size across the nine subfigures is also relative, with each venue marked with the same color. We place explicit labels on some of the larger bubbles, the venues with the most papers for each topic.

Unsurprisingly, the *RE conference* and *journal* are the most popular venues for all topics. *SAC* also makes a prominent appearance in 6/8 of our topics of focus. The general RE topic occurs most frequently in a wide variety of venues (Fig. 9a). We can see that papers involving agents or reasoning are more likely to appear in *ICSE*, compared to other topics, while papers on reasoning, NFR and softgoals, and Scenarios are slightly more likely to appear in *ER*. Papers on adaptation, variability, and evolution are more likely to appear in *REFSQ* or *MODELS*, while papers on conflicts, scenarios, or reasoning appear more often in *TSE*.

### RQ6: Citations

**Fig. 10:**
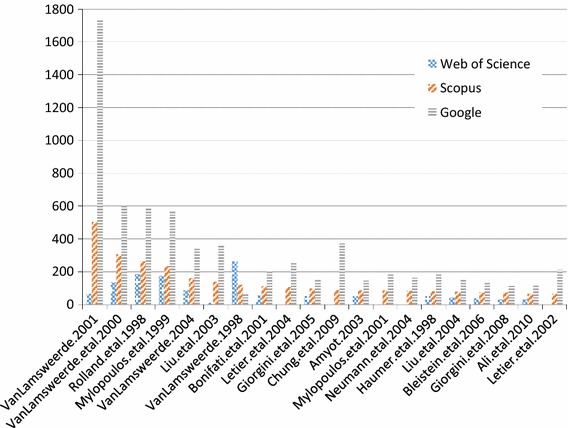
Top 20 cited GORE publications ranked according to Scopus citations, also showing Google Scholar and Web of Science citations (**RQ6(a)**)

**Fig. 11:**
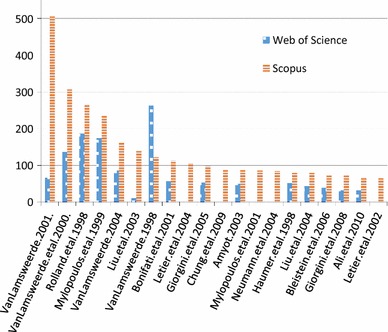
Top 20 cited GORE publications ranked according to Scopus citations (Google Scholar data removed) (**RQ6(a)**)

We show the top 20 cited papers as per Scopus in Fig. 10, answering **RQ6(a)**.^2^ We see that the Google Scholar citations for van Lamsweerde’s 2001 Guided Tour [44] dominates all other citations. Although this paper is also the most cited in Scopus, the differences between it and others are not as large, highlighting the different algorithm that Google uses to count citations. We show an alternative, more readable version omitting Google Scholar results in Fig. 11. Here we can see that there are a few highly cited papers, while citations for the other papers tail off gradually (**RQ6(b)**). We see this as a common phenomenon in a research area, where a few papers become seminal and are the default, “go-to” citation for an area. As mentioned in Sect. 4, 41% of the 966 papers had zero citations, and 616 out of 966 (64%) papers had less than three citations. Of these 616 papers, only 242 are recent, from 2013–2015. This means there are many older GORE-related papers which are not highly cited.

In general, these charts highlight the differences between citation sources (**RQ6(c)**). If possible, it is best to consider multiple sources of citations when analyzing publication data. In our case, we have collected all three data points, but focus on Scopus as a data source which is intermediate when compared to Google Scholar or Web of Science.

**Fig. 12:**
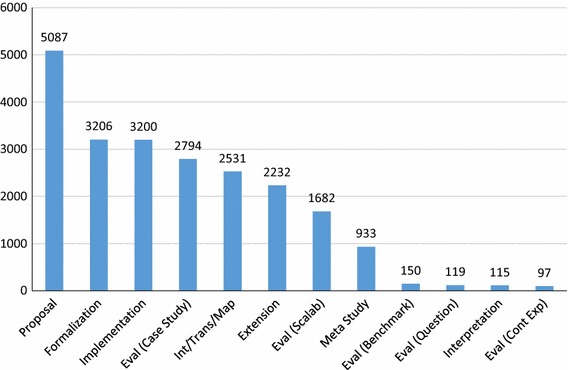
Citation per paper types (**RQ6(d)**)

We can look at which *types* of papers are most widely cited, answering **RQ6(d)**. To calculate the results for this research question, we sum the citations for all papers which have been tagged with a particular type. Recall that papers have multiple tags; thus, the sum of the citations will be larger than the sums in the previous charts. Figure 12 shows that proposals are by far the most cited type of papers. However, as almost all papers in our SMS (91%) proposed something new, this result is not surprising. Beyond this result, formalization and implementation are the next most cited types of papers, with case studies, integrations/transformations/mappings, and extensions close behind.

**Fig. 13:**
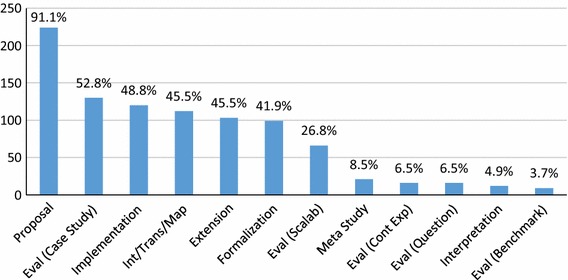
Total number of paper types in our SMS [for comparison in **RQ6(d)**]

We do not normalize the results via number of papers of these types included in our SMS, but one can compare to the occurrence of these paper types in general, aggregated from information in Sects. 5.1 and 5.2, in Fig. 13. We can see that relative to their occurrence, Formalizations are more highly cited, while Implementations are slightly more cited than the rate at which they occur. Case studies are cited at a slightly lesser rate than their occurrence, as are integrations/transformations/mappings. Not surprisingly, meta-studies (like this SMS) are cited at a much higher rate than their occurrence. We can make the initial conclusions that in GORE, formalizations, implementations, and meta-studies have the potential to make the most impact, relative to their numbers.

**Fig. 14:**
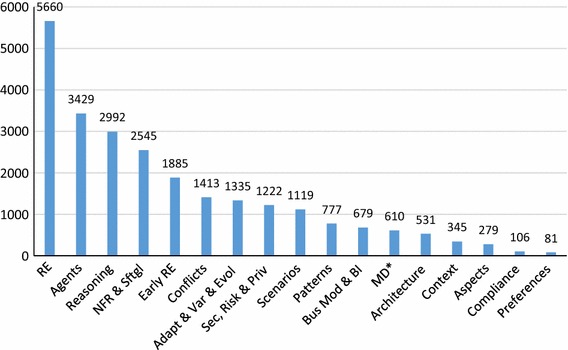
Top 20 most cited GORE topics (**RQ6(e)**)

We can also examine which of our identified *topics* are the most cited (**RQ6(e)**). We show the top 20 cited topics in Fig. 14. As expected, the most cited topic is requirements engineering (RE), followed by agents, systematic reasoning and non-functional requirements. We can see that beyond the general RE topic, no other topics stand out as obviously dominant, the slope of topic citations makes a gradual decline from agents to preferences. As one would also expect, the specificity of cited topics seems to increase as the number of citations decreases, i.e., agents and systematic reasoning are quite general topics, while other topics like compliance and preferences are more focused.

For comparison, one can compare Fig. 14 to Fig. 4, showing the total number of papers in our SMS with each topic tag. We do not see many significant differences between the trends in these figures, meaning the topics are cited with the same approximate frequency as papers concerning that topic are produced. One exception may be adaptation, variability, and evolution, which is number five in terms of number of papers but seven in terms of citations. This may be due to the relative newness of the topic, as shown in Fig. 5. Security, privacy, and risk, on the other hand, is the 11th most popular topic, but is eighth in terms of number of citations, potentially meaning that this particular sub-topic has more relative impact.

**Fig. 15:**
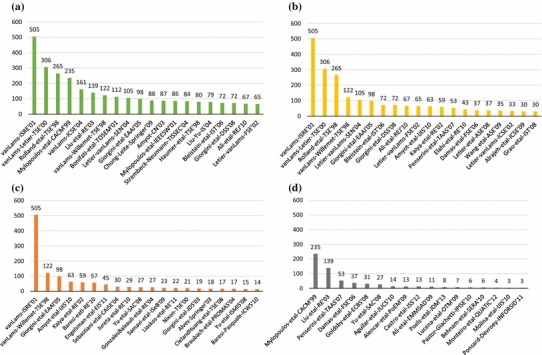
Dominant papers within selected topics (**RQ6(f)**). **a** Requirements engineering (RE). **b** Systematic reasoning. **c** Conflicts. **d** Model-driven development (MDD)

We have also asked whether there are dominant papers within each topic (**RQ6(f)**). When examining these data, we see that van Lamsweerde’s 2001 Guided Tour [44] dominates all other papers for about half of our topics. We show an example of a topic in which this paper clearly dominates, conflicts, in Fig. 15c. In other cases, the paper leads, but less dramatically, for example requirements engineering (RE) and systematic reasoning in Fig. 15a and Fig. 15b, respectively. In these topics, the second and third most cited papers are more highly related to the topic, i.e., are not survey papers.

Finally, there are some topics where this paper does not appear, for example model-driven development in Fig. 15d. In this case, the most cited paper is a general introduction to GORE from an object-oriented (OO) perspective, not explicitly focusing on MDD but emphasizing the link between goals and OO models. We see that authors tend to cite the most general papers, and these results support the previous conclusion that a few, rather general papers become seminal and are the default, “go-to” citation for that area. Charts for the other topics show similar trends.

**Fig. 16:**
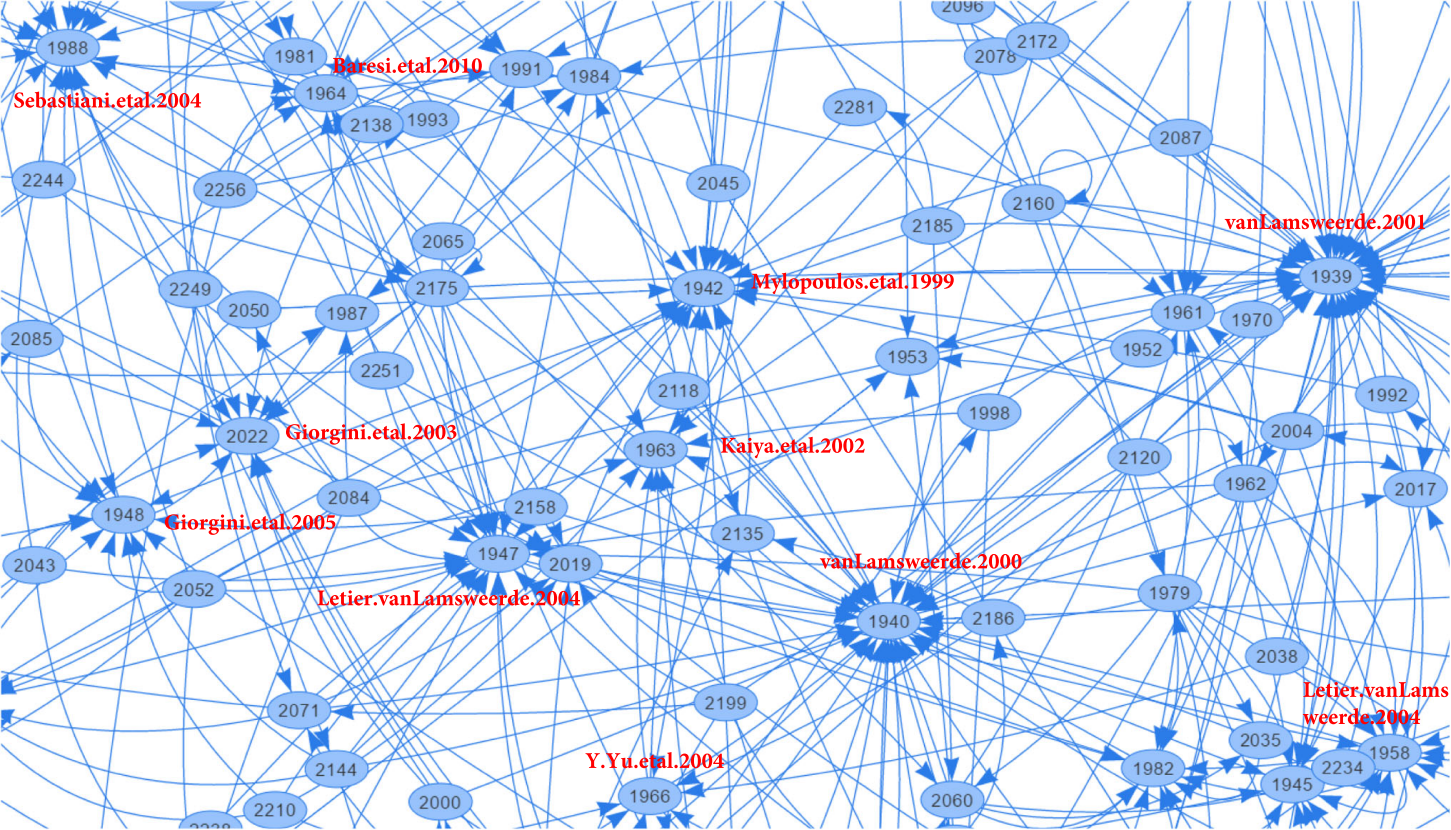
Excerpt of author citation network (**RQ6(g)**)

It is also interesting to look at the network of citations, to understand not only which papers are the most cited, but which papers have cited which other papers (**RQ6(g)**). To answer this question, we have created scripts which use the Scopus API to mine the citation data from Scopus [40], focusing on the 246 papers included in our SMS, in order to create a graph of citations. We can see a partial view of the core of this graph, with a few highly referenced papers labeled for illustration, in Fig. 16. Paper IDs here correspond to the paper IDs in our database, viewable in our full list of included papers. An interactive, full version of the citation graph can be found online.^3^ In this interactive version, hovering over a node shows the paper title, while clicking on a paper ID opens the paper’s corresponding page in Scopus.

Looking at the full version of the graph, we can see that 43 papers circle the outside edge of the main cluster, meaning that 17.5% of the 246 papers in our survey are not connected via citation to the main center cluster, and in terms of related work, these papers are isolated. There is another set of about 15 papers on the outside of the main cluster which are referenced by the cluster, but do not reference papers within the cluster. These papers are known to authors within the main core, but do not reference the core itself. The remaining 188 papers are at least minimally attached to the center cluster, many of which are strongly attached, indicating that there is a core, self-citing GORE community.

### RQ7: Authors

**Fig. 17:**
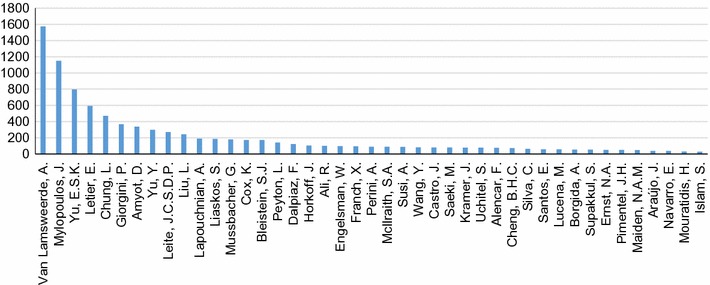
Top authors by total number of Scopus citations (**RQ7(a)**)

**Fig. 18:**
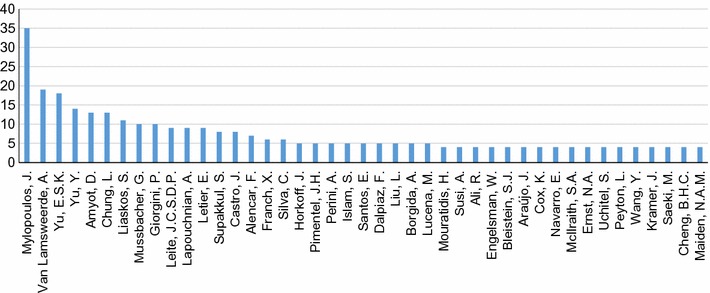
Top authors by total number of publications included in the SMS (cutoff >3 publications included) (**RQ7(a)**)

We evaluate the main contributing authors to GORE by examining the top counts for total number of citations via Scopus for included publications (Fig. 17) and total number of papers included in our mapping (Fig. 18), answering **RQ7(a)**. In terms of citations, the chart drops off fairly dramatically after the top five authors, showing a similar phenomenon as in **RQ6(a)**, where a few researchers are *the* researchers to cite when it comes to GORE. Figure 18 shows that numbers of included papers drop off somewhat more gradually, showing that the community is more inclusive when it comes to active members.

**Fig. 19:**
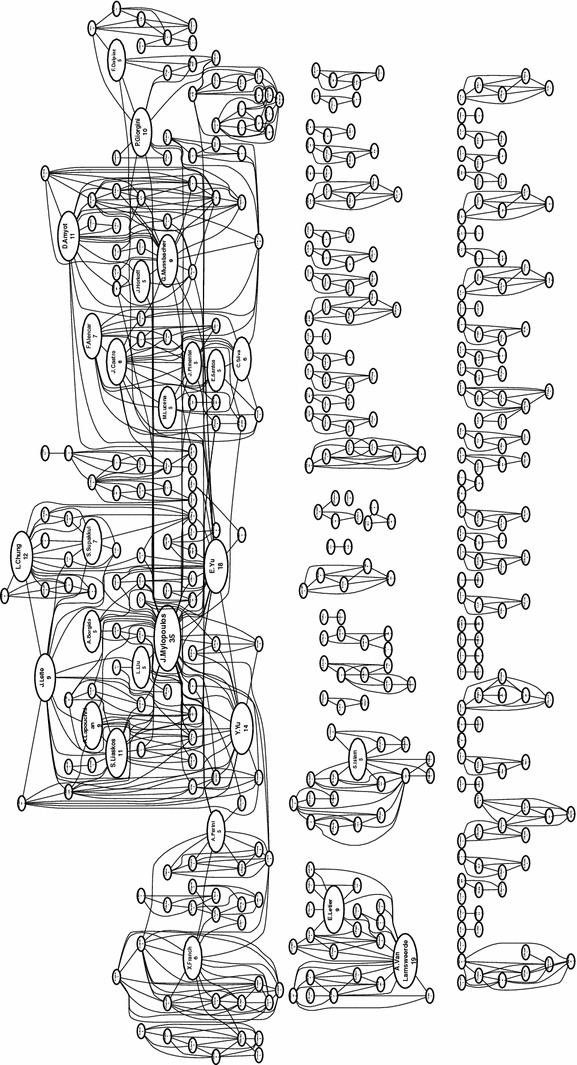
Full co-author network (**RQ7(b)**)

**Fig. 20:**
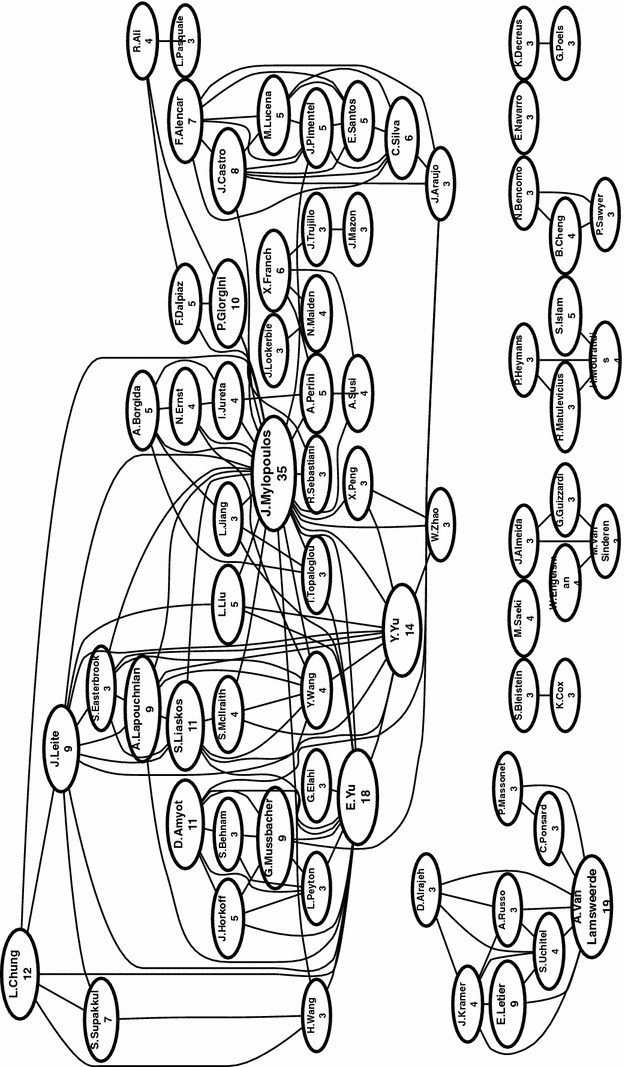
Co-author network showing authors with 3 or more papers included in the SMS (**RQ7(b)**)

We also examine the network of authors, i.e., which authors have written papers with which other authors included in our SMS, answering **RQ7(b)**. We present the full high-level view of the co-author network in Fig. 19, and a filtered view showing authors with more than 3 publications included in our SMS in Fig. 20. Figure 19 is also viewed online.^4^

In Fig. 19, one can note the presence of clusters, sets of co-authors. We can see one large cluster at the top, which can be further divided roughly into sub-clusters and several smaller clusters along the bottom. There is a total of 66 disconnected clusters, including 421 authors. The biggest cluster at the top includes 164 authors (39% of all authors), while the three largest clusters include 202 authors (48% of all authors). We have highlighted authors that have five or more publications included in our SMS, indicating the dominant member of the clusters. Note that the sizes of the corresponding circle for these highlighted authors are proportional based on their paper count, while all other authors have a fixed and small size (this is done to make the figure more readable).

We show a more readable version of the co-author network in Fig. 20, presenting the top 70 authors, each having three or more papers in the SMS. Here, the sizes of the author circle are proportional to their paper count. In this filtered view, we can note one large cluster at the top, with eight smaller clusters along the bottom.

From these figures, we can see generally see that collaboration within GORE relatively high, but still divided into various “camps.” These camps can be identified mostly by GORE framework, with the top large cluster focusing mainly on the NFR Framework, i*, Tropos, and GRL. The KAOS camp can be seen in the middle far left. It’s interesting to compare these figures to the citation networks in Fig. 16 and online. Here, the only clear camp is the core cluster of citations in the middle. In a broad sense, KAOS and i*-family authors cite each other, but do not co-author papers together.

### RQ8: General interest

When looking at overall interest in GORE, we can refer to Figs. 2, 3, and 5, each of which shows the total number of GORE papers per year included in our SMS in the top line of the chart. We can see that interest in GORE has risen significantly from 2008 to 2012. The recent drop could be because of the nature of our Scopus citation cutoff, or could be a genuine drop in interest.

## Summary and discussion

### Survey results

By analyzing the top-cited 246 papers as per Scopus, we have made several observations about the GORE field, enabling us to gain a high-level understanding of the progress made. We can observe some trends in research topics, notably a rise in adaptation/variability/evolution, but most of the popular topics seem to rise and fall with the number of papers. The KAOS and i* Frameworks continue to dominate nearly equally, with the majority of papers remaining non-committal when it comes to selecting a particular framework. Many (52) new frameworks have been introduced and not reused in subsequent publications. In terms of venue, RE and REJ dominate, but we can still see a wide variety of venues, with GORE publications spread in many publication areas. A small number of top authors and papers dominate in terms of citations, but the degree of involvement in terms of number of included papers is spread more widely. Overall, GORE has seen increased interest in recent years, possibly with a dip in interest more recently.

We see that the focus of GORE historically was to propose new methods, but more extensive use of past approaches, in the form of implementations, integrations and extensions, appears to be rising slightly. In terms of evaluation, about half of the GORE publications contain a case study, with this number appearing to increase slightly; scalability tests are still in use, while other forms of evaluation are rare. Still, in conjunction with our findings of many papers with low citations, we would hope to see even more convergence and utilization of existing work, instead of a steady stream of new proposals.

We can hypothesize that the proliferation of new idea papers may be due to both the complex nature of many RE problems and the maturity of the field. RE, as a research field, is relatively new and can be seen as a very rich research area with many difficult open problems that require complex new ideas as solutions. We can compare the scope and history of the field to other technical research areas. For example, databases can be seen to be narrower and more focused, and after a few new ideas papers by Codd [8], successive work has been largely evaluation, application, and innovation of industrial practice. On the other hand, AI is even broader than RE and so new ideas papers keep being produced. But it is also more mature in that there are more evaluations and applications to practice. Notice that both AI and databases are more than 20 years older than RE.

In terms of complexity, we believe that new ideas which are more complex, addressing harder problems are more likely to see extensions. It can be argued that understanding and evaluating the socio-technical divide between complex human organizations and complex systems is a particularly hard problem, which may be why the area of GORE research appears to have difficulty converging.

It is interesting to compare the trends in GORE to other RE research topics. Although this is challenging without similarly structured SMS for other topics, we are able to make some comparison regarding the use of empirical methods. An editorial by Daneva et al. evaluates the status of empirical methods in RE by looking at existing SLRs [9] and finds that the number of empirical studies published in RE venues has increased dramatically in the last ten years. In our results, although we note what appears to be a slight increase in case studies, we find the number of GORE-related papers with evaluation has increased more or less at the same rate of increase of GORE papers in general. In this light, GORE evaluation is increasing, but only relatively to the number of GORE publications. It is not clear whether this is also the case for RE in general, i.e., more evaluation studies because of more published papers in general.

Interestingly, although case studies are on the rise, their rate of citation is not proportional to their number, possibly indicating that authors are more likely to cite very technical contributions like formalizations and implementations. Although we may call as a community for more evaluation, we also need to be willing to cite and make use of such empirical papers.

Examining the communities of co-authors, we can see that the level of isolation in the co-author network is larger than in the citation network, meaning that most GORE papers do due diligence in their related work, but do not necessarily collaborate with the authors they cite. We expect that this is typical behavior in a particular research area, but are not aware of similar data sets for comparison.^5^

Although the co-author network shows much collaboration, we can see evidence of the divergence and isolation of various camps or schools. This divergence makes it challenging for potential new users to break into the area of GORE. Using goal models requires not only a knowledge of the basic concepts and motivations for goal orientation, but understanding of the differences between the various frameworks. In order to apply GORE, one is almost forced to “pick a side,” an unfortunate barrier to adoption. This may also help to explain why the most popular choice when using GORE in a paper (21.5%) is to use a general, unnamed goal modeling framework, instead of aligning to a particular existing camp. We hope to see more future convergence in the GORE community.^6^

For those planning to make future research contributions to GORE, we can use our data and analysis to make recommendations. (1) As the breadth of available GORE research is wide, due diligence is required to find related work. It is likely not enough to cite the “usual suspects,” but a more detailed literature search should be performed, making an effort to understand, adapt, extend, and reuse what has been done, instead of producing new proposals which may have a high-degree of overlap with existing work. (2) It would be ideal to see an increased focus on evaluation of existing methods, rather than the introduction of new ones. It would be even better to see these evaluations cited and used as a basis of further work. (3) Authors should aim to increase collaboration and convergence between different goal modeling camps. (4) Plain clear wording in the title, abstract, and keywords are important; both to be included in this and future meta-studies, but also to help future readers more easily pick up on the work.

### Survey method

In order to store and process a significant quantity of data, we designed an extendable and adjustable database schema for the publication reviews. The database technology was built with MySQL, with a front end in HTML and PHP, allowing us to view all papers and add information for particular papers. Furthermore, we adapted existing business intelligence techniques for SMS data analysis. The introduction of BI techniques provides several advantages: (1) data are analyzed in order to detect inconsistencies, thus improving data quality, (2) the system can generate reports and answer multiple questions with little effort, including others than those initially posed by the authors, and (3) data can be shared as a service or as an object so that other researchers can conduct their own studies and reach their own conclusions.

Following this perspective, we built an analytical database (data warehouse) parallel to the database supporting the SMS process. Data were then restructured and cleaned during the migration from the SMS database to the analytical database. As such, we can consider our analytic system as a layer built on top of the existing SMS database. We have used the method proposed in [31] in order to model SMS requirements and move from research questions to information entities that must be stored in our SMS data warehouse. We have used defined data dimensions to extract data from our databases corresponding to each of our research questions.

An alternative approach would have had us use one of the existing tools to support SLR [30]. Although this may have been helpful, at the time of the SMS design we were generally unaware of these tools. At the time we were comparing our potential tool support to existing tools supporting qualitative coding (e.g., [37]), and given the database expertise in our team, we preferred the flexibility of designing our own schema and interface.

Our initial conceptualization of the survey design was a “next-generation survey,” focusing on the evolution and maturity of particular ideas, going beyond the current state-of-the-art in empirical software and requirements engineering following the systematic process laid out by Kitchenham et al. [27]. Maturity could be defined by phases similar to those reflected in our paper types (proposal, formalization, implementation, evaluation, etc.), but the real measure of maturity could be gained by looking at the references for each paper to determine how the reference relates to the current work: does the paper extend, evaluate, map to, implement, formalize, etc., the related work, or does it just mention the other paper for the purposes of comparison, rather than an advancement of ideas?

We set the initial design of our survey along these lines, creating a classification scheme for references between papers. However, after two rounds of development and ICR testing, we found that agreement among coders for these reference classifications was too low, with an average of 0.14 after round two, far lower than our level of agreement for paper types and topics, even though the classification schemes for paper types and references were similar. These results in and of themselves are interesting. This tells us that (1) authors are not particularly good at making the relationship between their own work and other publications clear, and (2) coders, even after much practice and discussion, are not sufficiently good at being able to classify the relationships between papers accurately. Furthermore, the process of coding references was incredibly time consuming, explaining why our ICR process of coding 55 papers took six months, while coding about 60 papers, twice, without considering references took us only about two months.

Issues of disagreement among coders and feasibility of getting objective answers also played an important role in determining the research questions that we tried to answer in the first place. Of course, it would be interesting to try to answer questions about the root causes of the trends we have discovered in this survey. But such questions are hard to answer in an objective fashion for lack of information, and/or because they were too subjective. Accordingly, we chose research questions on the basis of how interesting they were and also whether we had the means to answer them objectively, on the basis of available data, as opposed to opinions of coders. This is consistent with the spirit of evidence-based software engineering. This suggests that the state of the art in evidence-based software engineering needs to be advanced so that we have techniques and tools for answering deeper questions about trends and root causes for a research area under review.

The initial survey design had us using snowballing as the primary means of finding papers, in line with the focus on references and idea evolution in the next-generation survey. Once we decided to abandon the next-generation survey design and focus on a traditional mapping study, we adjusted our design to find candidate papers via systematic search. Existing work argues that snowballing is in fact a more appropriate technique to find candidate papers, due to the challenges in finding a search string which covers divergent terminology [51].

We agree that in the case where the set of papers to be found is reasonably small, e.g., 50–100 papers, that a combination of snowballing and systematic search may be optimal. The problem in this case is that the set of GORE-related papers is very large. Our systematic search results returned 966 results, of which we examined 350 in detail, including 246 papers (about 2/3). From this, we can roughly extrapolate that there are at least 700 GORE-related papers (2/3 of 966).

In a previous SMS, we^7^ snowballed over papers related to a sub-topic within GORE, covered a similar number of papers as in this submission (246), without converging in our snowballing process. We had to stop by setting a snowballing depth limit (of two), and even so the reference network exploded so quickly we could not complete the process. In this case, we supplemented the process with systematic search. Similarly, in our first attempt at paper finding for this SMS, the snowballing process exploded—after 110 papers we found little sign of convergence. Considering our experiences and the large set of relevant papers, we had a choice of performing systematic search with some cutoff, in this case the number of citations, or snowballing with some cutoff, likely snowballing depth. Both will produce incomplete sets, but the former method, in our opinion, produces an incomplete set of papers in a more objective way, focusing on the most cited papers. Future work could supplement our findings with snowballing, producing a more complete (but possibly more subjective) set.

In this work, we have taken a bottom-up approach to understanding the space of work in GORE. One could also take a complimentary top-down approach, mapping the categories extracted from our SMS to existing RE-related ontologies or taxonomies (for example, in [5] or, more specific to GORE, in [44]). The first step would be to find candidate ontologies and taxonomies and to assess their degree of consistency with our discovered categories. We suspect that the process of mapping and evaluation, although interesting, will not be trivial. Thus, we leave this step to future work and invite others to use our categories in such analysis.

## Threats to validity

We can identify several threats to the validity of our study as per [49]. Although we have covered 246 papers, we have omitted those papers with less than 3 citations as per Scopus, as well as workshop papers, threatening *conclusion validity*. By omitting workshop papers, we may miss influential work in the area, or work published in workshops which later became conferences. Given that this is a first general mapping of goal model work, we focus on those publications which are more rigorously peer-reviewed. Furthermore, by using the citation cutoff of three, we put greater emphasis on older work, discounting new work in the area which may not yet be extensively cited.

The inclusion or exclusion of papers in our survey may be subjective or error prone, i.e., does a paper involve GORE significantly? However, we mitigate this threat by having two people check the inclusion/exclusion of papers and by discussing borderline papers.

It would have been desirable to have a more extensive set of topics. After creating the initial set of topics, we found several further topics which were candidates for inclusion (e.g., scenarios). A few of these topics were included after discussion and an agreed-upon definition; however, we avoided adding many new topics as we had already conducted our ICR tests and could not guarantee the reliability of further topics.

Our systematic search criteria may also be subject to critique, threatening *construct validity*. As many scientific papers talk about the “goal” of the paper and have some sort of scientific model, searching for variants of “goal model” (e.g., “goal modeling,” “goal modeling”) provides too many false positives, likewise when replacing goal with its synonyms, e.g., intention, motivation. Thus, we chose to use only variants of “goal modeling” without using synonyms of goal and to include “requirements” in our search. We also experimented with the use of “engineering” in our query, but found the results too narrow. Although we arrived at what we believed was an effective search string, we may miss papers which concern GORE but do not use variations of “goal model” in the title, abstract, and keywords. A notable case is Yu’s RE’97 paper [50], winning a most influential paper in RE’07, but not caught by our systematic search string as it does not explicitly use the terminology in our search string. This emphasizes the importance of keywords when writing papers, although in the case of [50] the terminology for GORE had not yet converged to recognizable keywords. Future work could expand our data using snowballing.

We have discussed extensively our coding process and measurement of ICR, relating to the *Internal Validity* of our results. We hoped that our ICR measure would be higher, particularly for paper topics, but given the challenges of qualitative coding, we accept these results. Given the large number of coders we had and the large number of categories, it was particularly challenging to achieve high agreement scores.

The authors of this study have significant experience in goal modeling, typically with i*-related languages. This may bias survey tagging, threatening *external validity*. It is possible that coders with a different background would find different codes, or, if their background is particularly diverse, would have trouble converging and agreeing on codes. In a way, our similar backgrounds made a sufficient level of agreement possible. However, we believe these issues are not unique to us, but shared by all those who conduct SMSes. Furthermore, much of our tagging process was performed at a high level of abstraction (e.g., does a paper use i* or KAOS?) not including an evaluation of paper quality, helping to mitigate validity threats which may arise from our common background. Several authors of this study are authors of publications included in the SMS. However, as candidate publications were found via systematic search and objective citation data, we mitigate these threats.

Relating to external validity, with any systematic literature review, it is important to demonstrate sufficient *repeatability*. If another set of people were to reclassify the same group of publications using our tags, we have confidence that our tag definitions would help them make choices which are fairly consistent with our results. However, outsiders could not be present for our extensive discussions, and it is not feasible to make collected group knowledge explicit. Furthermore, if another group went through a different process of grounded paper topic building, as described in Sect. 4.1, they may come up with a different set of topics. This is an unavoidable side effect of qualitative coding; nevertheless, we believe our results provide a useful contribution to the RE community. We make all of our survey data publicly available^8^ and welcome further analysis, including alternative codes.

## Related work

*Literature reviews in SE* We have created our SMS by adopting the methods and approaches prescribed by Petersen et al. [34], specifically focusing on a map of available work, rather than a detailed survey evaluating publication quality, clearly defining our process of finding and including papers, making our research questions clear. As our survey is designed as an SMS and not an SLR, we do not perform a deeper evaluation of paper quality, for example using criteria provided by Ivarsson and Gorschek [22]. In the trade-off between paper volume and survey depth, in choosing to conduct a SMS, we focus on volume, covering many papers in a shallow way. Future work should evaluate GORE literature, likely covering smaller sets of publications, in more depth.

Kitchenham et al. provide guidelines for SLRs in software engineering. When applicable, we apply many of these guidelines to our SMS, including clearly specifying a hypothesis (in our case research questions), defining populations (publications from systematic search of Scopus), defining a process, providing raw data, and making extensive use of graphics [24, 25].

Work by Pham et al. focuses on a social network analysis of computer science publications, investigating collaboration and citations [35], applying such analysis to the CAiSE conference series in [23]. Our analysis of GORE citation networks (**RQ6(g)**) and the network of co-authors (**RQ7(b)**) bears similarity to this work; however, we did not go into a detailed analysis of the relationship of the GORE community to other communities, or the evolution of our connectivity. Future efforts could use our data for this type of analysis.

### *Meta-reviews in RE*

 Bano et al. perform a meta-review of systematic literature reviews in RE, finding that the number of systematic literature reviews in RE has increased dramatically from 2006 to 2014, but that the quality of such studies has decreased. They measure quality by looking at inclusion/exclusion criteria, search space adequacy, quality assessment of primary studies, and information regarding primary studies. A mapping study, by nature, does not evaluate the quality of or provide specific information regarding primary sources [26, 34], so we believe the latter two quality categories do not apply to this study (or other SMEes in RE). Regarding the first two points, we listed our inclusion/exclusion criteria in Sect. 3 and have selected Scopus as our publication source as it covers major databases in our field (IEEE, Springer, ACM) avoiding the need to combine results of multiple databases.

The Daneva et al. evaluation, looking at existing SLRs in RE [9], finds two reviews related to GORE [13, 20]. The former is the previous work of some of the authors, while the latter, focusing on compliance, was omitted from our survey as it appeared in a workshop.

### *GORE literature reviews*

 Our survey found 21 papers marked as meta-studies, a tabular summary is found in Table 8. Note that it appears that the two Teruel et al. papers ([42, 43]) are likely the same paper, but the paper appears twice in Scopus under two different names and was therefore reviewed twice in our SMS. It is interesting to note that although meta-studies make up 8.5% (21/246) of our papers, their total citations according to Scopus accounts for 15.7% (933/5941) of total citations for all included papers. Thus, as expected, meta-study papers are more highly cited when compared to other types of papers, although this relative increase is actually rather small. We also find that most of the prominent GORE-related literature reviews were not performed systematically, with a few exceptions.

The most cited GORE literature review (also the most cited GORE paper) is van Lamsweerde’s guided tour of the area as per 2001 [44]. This work motivates the use of goal orientation and summarizes existing methods for modeling, specifying, and reasoning over goals. Chung and Leite review the state of the art in non-functional requirements, exploring definitions, classifications and representations of NFRs, reviewing prominent publications at the time [6]. The paper associated with van Lamsweerde’s RE’04 keynote [45], provides an overview of work relating primarily to the KAOS framework. Van Lamsweerde and Letier further motivate the need for GORE by enumerating the limitations of object-oriented approaches as applied to solving the requirements problem [47]. In doing so, they review several approaches both within and outside of GORE. In addition to presenting the goal/strategy map, Rolland and Salinesi perform an extensive overview of GORE as per 2005 [39].

In [1], Amyot and Mussbacher perform a SLR of publications, finding 281 publications using the user requirements notation [containing the Goal-oriented Requirement Language (GRL)]. The focus of our current survey is broader and more shallow, looking at all GORE notations, including GRL, and not getting into extensive details.

Further work covers GORE with a more narrow focus. Grau et al. compare and contrast six dialects of i* [14], while Regev and Wegmann review GORE methods in order to improve definitions of goal types using principles from regulation mechanisms [38]. Otto and Antón include goal modeling techniques related to law in their survey of RE and legal requirements [33], while Decreus et al. look at six techniques transforming i* to business process models [11]. Mussbacher et al. provide an extensive qualitative comparison of the Aspect-oriented User Requirements Notation (AoURN) to URN and other aspect-oriented techniques, including scenario-based techniques and other goal-oriented techniques [32].

**Table 8:** List of meta-study papers included in our SMS

Title	Authors	Venue	Year	Scopus citations
Goal-oriented requirements engineering: A guided tour	VanLamsweerde, A.	RE	2001	505
On non-functional requirements in software engineering	Chung, L., Leite, J.C.S.D.P.	ER	2009	88
Goal-oriented requirements engine ring: A roundtrip from research to practice	Van Lamsweerde, A.	RE	2004	59
Addressing legal requirements in requirements engineering	Otto, P.N., Antón, A.I.	RE	2007	52
User requirements notation: The first ten years, the next ten years	Amyot, D., Mussbacher, G.	J. of Soft.	2011	46
Modeling goals and reasoning with them	Rolland, C., Salinesi, C.	Eng. and Managing Soft. Reqs.	2005	25
A comparative analysis of i*-based agent-oriented modeling languages	Ayala, C.P., Cares, C., Carvallo, J.P., Grau, G., Haya, M., Salazar, G., Franch, X., Mayol, E., Quer, C.	SEKE	2005	25
Reasoning about alternative requirements options	Van Lamsweerde, A.	Concept. Mod.: Found. and Appl.	2009	19
Where do goals come from: The underlying principles of goal-oriented requirements engineering	Regev, G., Wegmann, A.	RE	2005	18
Requirements modeling with the Aspect-oriented User Requirements Notation (AoURN): A case study	Mussbacher, G., Amyot, D., Araújo, J., Moreira, A.	TAOSD	2010	16
Analyzing goal models: Different approaches and how to choose among them	Horkoff, J., Yu, E.S.K.	ACM SAC	2011	14
Comparison and evaluation of goal-oriented satisfaction analysis techniques	Horkoff, J., Yu, E.S.K.	REJ	2013	13
From object orientation to goal orientation: A paradigm shift for requirements engineering	Van Lamsweerde, A., Letier, E.	CAiSE	2004	13
Research review: Investigating goal-oriented requirements engineering for business processes	Poels, G., Decreus, K., Roelens, B., Snoeck, M.	JDM	2013	8
A comparative of goal-oriented approaches to modeling requirements for collaborative systems	Teruel, M.A., Navarro, E., López-Jaquero, V., Montero, F., González, P.	ENASE	2011	7
Practical challenges for methods transforming i* goal models into business process models	Decreus, K., Snoeck, M., Poels, G.	RE	2009	6
Requirements evolution and what (research) to do about it	Ernst, N.A., Mylopoulos, J., Wang, Y.	EIS	2009	5
Comparing the comprehensibility of requirements models expressed in Use Case and Tropos: Results from a family of experiments	Hadar, I., Reinhartz-Berger, I., Kuflik, T., Perini, A., Ricca, F., Susi, A.	IST	2013	4
Using business goals to inform software architecture	Clements, P., Bass, L.	RE	2010	4
Comparing goal-oriented approaches to model requirements for CSCW	Teruel, M.A., Navarro, E., López-Jaquero, V., Montero, F., González, P.	ENASE	2011	3
Requirements engineering in the development of multi-agent systems: A systematic review	Blanes, D., Insfran, E., Abrahão, S.	ER	2009	3

Several meta-studies focus specifically on GORE reasoning. Van Lamsweerde discusses the work of Mylopoulos and colleagues concerning goal reasoning in [46]. Horkoff and Yu provide surveys covering GORE reasoning. The authors broadly cover many approaches in [21], including information required, methods used, and guidance for selection. In [17], a more narrow-focused survey is conducted, providing a comparative analysis of seven i*-related analysis procedures, exploring differences in results.

Poels et al. provide a SLR focusing on GORE for supporting business process modeling and management activities (GORE-for-BP), providing an overview of 19 available methods, and evaluating method maturity [36]. The authors make meta-models of the methods in order to understand and summarize existing approaches. Overall, they find divergence in modeling approaches and evaluate most approaches to be immature due to incompleteness, including vagueness in the transformation activities, method guidelines, and lack of validation.

Blanes et al. look at the application of RE techniques to multi-agent systems, performing a systematic literature review covering 58 papers in depth [4]. Sixty-nine percent of the techniques found were based on GORE. In terms of validation, the authors found that 64% of papers used case studies while 5% used controlled experiments, comparable to our results of 53% and 7%, respectively.

Several papers provide meta-studies in the form of experiments comparing various goal modeling frameworks. Teruel et al. perform a comparative evaluation of the NFR Framework, KAOS, and i*, specific to their capabilities in supporting collaborative systems [42, 43]. The authors apply each technique to modeling awareness requirements for Google Docs and then evaluate each framework based on a list of desirable features for collaborative systems, e.g., awareness representation, traceability, and supporting quantitative model. Their final quantitative scores rank i*, KAOS, and NFR as 5, −2, and −21, respectively. Hadar et al. compare the comprehensibility of requirements expressed as Use Cases versus Tropos (a GORE framework) using experiments [15]. Participants were asked to map text to requirements, understand, and modify models. Results with 79 students show that Tropos was more comprehensible, but also more time consuming.

Some meta-studies captured in our SMS are goal-oriented and contain a meta-study, but not necessarily a meta-study directly related to GORE. In [7], Clements and Bass present a lightweight GORE method (Pedigreed Attribute eLicitation Method, or PALM) which uses canonical business goals to elicit specific business goals, feeding into the architecture of software. The method uses a structured, but non-graphical syntax for expressing goals. In order to derive their canonical set of business goals, the authors perform a structured literature review focusing on business goals/models, extracting business goals, then clustering the results into ten categories.

Several papers propose some new contribution, but also include an extensive literature review. Ernst et al. review approaches related to requirements evolution as per 2009, including approaches for software, system, process, and requirements evolution, management, and traceability. Most work covered is not in the GORE area, but the authors use this broader review to frame their goal-based framework for monitoring and diagnosis, presented in the same paper [12].

As described in Sect. 1, our previous SMSes and SLRs have focused on GORE publications describing transformations/mappings, in order to understand how goal models can lead to downstream development [18, 20]. Although the RQs and inclusion/exclusion criteria of these publications bear similarities to the current work, the set of papers reviewed is different, as is the method used for finding the papers (systematic search with a citation cutoff vs. snowballing and systematic search with no citation cutoff).

## Conclusions and future work

We have provided the first general systematic survey of GORE, covering the 246 most cited publications, according to Scopus. We have chosen to give an overview of the field using a SMS, with an emphasis on descriptive graphics. We have focused our inquiries with a number of specific research questions, and used our results to make general recommendations for future GORE-related research. In the name of repeatability and enhancing the knowledge of the field, we have made our publication data and category descriptions publicly available.^9^ We encourage further analysis, investigation, and expansion of our data.

We remain interested in the next-generation survey concept, focusing on categorizing the relationships between publications to track the evolution of ideas. However, the difficultly in reliably tagging references between papers needs to be worked out, perhaps through some form of cooperative open tagging. Future work should investigate this and other possibilities.

## Footnotes

1 http://www.cs.toronto.edu/goreslm/.

2 Full paper references can be found here: http://www.cs.toronto.edu/goreslm/Papers.html.

3 http://www.cs.toronto.edu/goreslm/CitationNetwork.html. Please give time for the graph to load.

4 http://www.cs.toronto.edu/goreslm/RQ7_b1.pdf.

5 Work by [23] creates a similar co-author network for the CAiSE conference, but focuses on co-authorship and not citations for a much broader community.

6 The authors acknowledge that they are mainly from the i*/Tropos camp and are therefore currently part of the problem.

7 A subset of the authors.

8 http://www.cs.toronto.edu/goreslm/.

9 http://www.cs.toronto.edu/goreslm/.
